# Engineered self-assembling monolayers for label free detection of influenza nucleoprotein

**DOI:** 10.1007/s10544-015-9951-z

**Published:** 2015-04-11

**Authors:** Anton P. Le Brun, Andrei Soliakov, Deepan S. H. Shah, Stephen A. Holt, Alison McGill, Jeremy H. Lakey

**Affiliations:** Bragg Institute, Australian Nuclear Science and Technology Organisation, Locked Bag 2001, Kirrawee DC, NSW 2232 Australia; Institute for Cell and Molecular Biosciences, The Medical School, Newcastle University, Framlington Place, Newcastle upon Tyne, NE2 4HH UK; Orla Protein Technologies Ltd, Biosciences Centre, International Centre for Life, Times Square, Newcastle upon Tyne, NE1 4EP UK; Fujifilm Diosynth, Belasis Avenue, Billingham, Cleveland TS23 1LH UK

**Keywords:** Protein engineering, Surface plasmon resonance, Quartz-crystal microbalance, Neutron reflectometry, Self-assembled monolayer, Influenza

## Abstract

Integrating nanotechnology into useable devices requires a combination of bottom up and top down methodology. Often the techniques to measure and control these different components are entirely different, so methods that can analyse the nanoscale component *in situ* are of increasing importance. Here we describe a strategy that employs a self-assembling monolayer of engineered protein chimeras to display an array of oriented antibodies (IgG) on a microelectronic device for the label free detection of influenza nucleoprotein. The structural and functional properties of the bio-interface were characterised by a range of physical techniques including surface plasmon resonance, quartz-crystal microbalance and neutron reflectometry. This combination of methods reveals a 13.5 nm thick engineered-monolayer that (i) self-assembles on gold surfaces, (ii) captures IgG with high affinity in a defined orientation and (iii) specifically recognises the influenza A nucleoprotein. Furthermore we also show that this non-covalent self-assembled structure can render the dissociation of bound IgG irreversible by chemical crosslinking *in situ* without affecting the IgG function. The methods can thus describe in detail the transition from soluble engineered molecules with nanometre dimensions to an array that demonstrates the principles of a working influenza sensor.

**Electronic supplementary material:**

The online version of this article (doi:10.1007/s10544-015-9951-z) contains supplementary material, which is available to authorized users.

## Introduction

Self-assembly is the most effective route to the creation of complex nanoscale components and, by exploiting specific non-covalent interactions, highly ordered structures can be formed (Fletcher et al. 2013; Yu et al. 2014). In some cases these supramolecular assemblies need to be integrated into microscale sensors and actuators. In this paper we address the creation of a highly efficient immunosensor by integration of top down manufacture and bottom up self-assembly of engineered proteins. Whilst the surface immobilisation of antibodies provides the opportunity to create biosensors and other targeted nanoscale diagnostic devices, the immobilised components can be either randomly arranged or oriented for optimal antigen capture (Trilling et al. 2013). The simplest way to immobilise an antibody is through physical adsorption to a surface such as silicon. In the case of adsorption of immunoglobulin G (IgG) to silica surfaces, the IgG will adsorb with a flat-on orientation with some overlap of IgG molecules at higher surface coverage (Xu et al. 2006a). With physical adsorption of IgG, antigen can still be bound to the antibody, even with an albumin blocking agent co-adsorbed (Cowsill et al. 2014). However, the theoretical maximum binding ratio is rarely achieved, particularly at high antibody surface coverage (Xu et al. 2006b, 2007). On the other hand, oriented antibody immobilisation enhances antigen binding as all the binding regions face away from the surface, increasing the potential to capture antigen (Huy et al. 2011; Le Brun et al. 2011a; Liu et al. 2012; Shen et al. 2011). Orientation thus increases the signal per unit area allowing the device size to be minimised. Oriented immobilisation is usually *via* an intermediate protein immobilised to the surface which binds the antibody through a domain outside the antigen binding region (Trilling et al. 2013). Oriented binding has also been achieved by other methods such as immobilisation to a surface *via* the polysaccharide chain on IgG (Ho et al. 2010).

When creating biomimetic surfaces to integrate into microelectronics the surface that the biomolecules are to be immobilised to has to be considered. Gold is an attractive option as the surface is stable, conductive (Cornell et al. 1997) and amenable to label free techniques such as surface plasmon resonance (SPR) (Skottrup et al. 2008), surface acoustic waves (SAW) (Kogai et al. 2010), and quartz crystal microbalance with dissipation (QCM-D) (Amano and Cheng 2005) providing a rapid and sensitive form of detection. Gold also allows for self-assembly, the spontaneous deposition of a defined molecular layer by chemisorption. Molecules that self-assemble usually have a chemical group that will interact strongly with the surface allowing for the assembling molecules to orient themselves in a particular direction. Due to the strong interaction between the assembling molecule and the surface all the surface available for assembly becomes occupied promoting the formation of a dense monolayer. The chemistry for creating self-assembled monolayers on gold is well understood and relatively straightforward (Dubois and Nuzzo 1992; Love et al. 2005). Thiol-alkanes with functional groups at the alkyl terminus can be adsorbed onto gold surfaces through the thiol group adsorbing to the gold surface to form self-assembled monolayers. For example, polyethylene glycol terminated thiol-alkanes have be used to create surfaces that are resistant to protein adsorption (Prime and Whitesides 1993).

Previous publications have described the development of systems where outer membrane proteins from *Escherichia coli* can be used as engineered protein scaffolds on solid supports (Cisneros et al. 2006; Holt et al. 2009; Keegan et al. 2005; Terrettaz et al. 2002). The transmembrane section (residues 1 to 171) of outer membrane protein A (tmOmpA) has been engineered such that it can be immobilised to gold surfaces in a specific orientation through a single cysteine residue (Shah et al. 2007). The tmOmpA was then circularly permutated in extracellular loop 1 to create ctOmpA which acts as a scaffold to which functional domains from other proteins can be fused (Le Brun et al. 2011a). For example the ctOmpA scaffold has been fused to a single or double immunoglobulin-binding Z domain (ZctOmpA & ZZctOmpA) from *Staphylococcus aureus* protein A for antibody binding and was used to build arrays with bound IgG for antigen sensing (Le Brun et al. 2008, 2011a, b). However, whilst the ZZctOmpA arrays bound rabbit IgG tightly, they worked poorly for mouse monoclonal IgG which are the preferred component of many medical tests (Le Brun et al. 2011a).

Protein G from *Streptococcus* group C or G is a multi-domain cell wall-associated protein that binds IgG and human serum albumin. The B domain (herein referred to as protein G), which binds IgG, has an architecture consisting of a single α-helix packed into a three-stranded β-sheet (Gallagher et al. 1994). Whilst protein G and protein A have no sequence or structural homology (Tashiro and Montelione 1995), both compete for the same binding site on the constant (Fc) region of IgG, leaving the variable antigen-binding (Fab) region free to bind antigen (Sauer-Eriksson et al. 1995). Despite proteins A and G competing for the same Fc binding site, protein G binds more subclasses of IgG (Akerström et al. 1985; Björck and Kronvall 1984). This is likely to arise from the difference in the protein-protein interaction between protein G and protein A to Fc. The protein A-Fc interaction is mainly between hydrophobic residues whereas that of protein G mainly consists of salt bridges and hydrogen bonds between polar residues (Sauer-Eriksson et al. 1995).

Protein G and Protein A have been used as IgG capture molecules in immunosensor devices fabricated using self-assembled monolayers (Makaraviciute and Ramanaviciene 2013; Trilling et al. 2013). The IgG capture molecules have been immobilised to surfaces using self-assembled monolayers of thiolalkanes functionalised with carboxylic acid groups for amine cross-linking (Jyoung et al. 2006), nitrolotriacetic acid (NTA) for binding proteins with a hexa-histidine tag (Roth et al. 2013), or for binding biotinlyated proteins (Jung et al. 2006). Monolayers of proteins A and G have been formed by direct assembly onto gold by incorporating thiol groups (Oh et al. 2004), or through the use of fusion proteins such as glutathione *S*-transferase (GST) (Ha et al. 2006) or elastin (Gao et al. 2006). This work describes an array of ctOmpA protein G fusions designed to reduce the dissociation of mouse monoclonal IgG. Two protein G domains were fused to the N-terminus of ZctOmpA to create the array protein GGZctOmpA. Once the GGZctOmpA is immobilised to the gold surface the monolayer is completed with a thiol-terminated amphiphile with a poly(ethylglycol) head group. This amphiphilic filling molecule supports the array protein and reduces subsequent non-specific binding (Cisneros et al. 2006; Le Brun et al. 2011a; Terrettaz et al. 2002). The GGZctOmpA array is designed for the label free detection of protein analytes in clinical or veterinary samples and when loaded with a monoclonal IgG the array becomes highly specific for the binding of defined epitopes of the target antigen. This specificity in turn assembles oriented antigen allowing a second different monoclonal antibody to target another epitope on the other face of the antigen allowing a “sandwich assay” of high sensitivity to be created. The target antigen in this work is the influenza nucleoprotein (NP). NP forms oligomers that encapsulate the genomic RNA and the RNA polymerase of the influenza virus in ribonucleoprotein particles (Coloma et al. 2009; Ruigrok et al. 2010; Ye et al. 2006). The nucleoprotein, highly conserved between strains and type specific for influenza A and influenza B, has been a preferred target protein for influenza detection over the highly variable surface glycoproteins haemagglutinin and neuraminidase (Farris et al. 2010). This paper describes the *in situ* physical characterisation of the entire assembly process using surface plasmon resonance, quartz crystal microbalance with dissipation and neutron reflectometry.

## Materials and methods

### Materials

Antibodies were purchased from HyTest Ltd (Turku, Finland). Molecular biology and protein purification materials were purchased from Invitrogen, Novagen and GE Healthcare (UK). D_2_O was purchased from CDN Isotopes (Canada). Cross-linking reagents were purchased from Pierce Scientific (Cramlington, UK). All other materials were purchased from Sigma-Aldrich unless otherwise stated.

### Construction of expression plasmids

DNA digestions, ligations and transformations were carried out using standard molecular biology techniques (Sambrook and Russell 2001). The GGZctOmpA protein was constructed by designing a gene encoding a tandem pair of B-domains from protein G and ligating this to a scaffold protein (ZctOmpA) that contains a single N-terminal Z-domain fused to a circularly permuted tmOmpA scaffold (Le Brun et al. 2011a). This construct also contains a His_6_-tag at the N-terminus.

The amino acid sequence of influenza A nucleoprotein (NP) was obtained from the NCBI Influenza Resource website (Bao et al. 2008), reverse translated and codon optimised to provide a DNA sequence for expression in *E. coli*. The gene was synthesised by Epoch Biolabs (Texas, USA) and subsequently ligated into a pET3d-based expression vector that incorporates a His_6_-tag at the N-terminus of the expressed protein. This protein is referred to as recombinant NP (rNP).

### Protein expression and purification

#### GGZctOmpA

GGZctOmpA was expressed and purified as described previously for ZZctOmpA (Le Brun et al. 2011a) and a detailed description is included in the supplementary information. Refolding of the GGZctOmpA protein was confirmed by Circular Dichroism spectroscopy (Supplemental Fig. S1).

#### Recombinant influenza A NP protein

Expression of rNP was carried out in *E. coli* BL21-AI cells in LB broth and was induced with 0.2 % L-arabinose. The rNP was purified from the cell lysate by immobilised metal affinity chromatography followed by cation exchange chromatography. The purified protein was analysed by SDS PAGE to ensure >95 % homogeneity of rNP (~57 kDa). The binding of anti-Influenza A NP mouse monoclonal IgGs mAb245 and mAbA108 (HyTest) was confirmed by Western blotting (data not shown). A detailed description of the expression and purification of rNP can be found in the supplementary information.

#### Deuterated proteins

Deuterated proteins were produced as described for GGZctOmpA but the media used was Silantes OD ^2^H rich growth media (Silantes, Munich, Germany) which is a rich media containing D_2_O for deuterium incorporation into proteins. Mass spectrometry analysis to determine the level of deuteration was carried out by the Sydney University Proteome Research Unit. A tryptic digest of protein from a gel slice of an SDS-PAGE band corresponding to the molecular weight of the protein was analysed by MALDI-TOF mass spectrometry. The amount of deuteration was determined by comparing the mass of the peaks from each fragment of the hydrogenous protein with that of the corresponding peaks in the deuterated protein (Supplementary Table S1).

### Surface plasmon resonance (SPR)

SPR experiments used BIAcore Au sensor chips in a BIAcore X100. The Au chip was cleaned by using piranha solution (3:1 H_2_SO_4_/H_2_O_2_ by volume) on the gold surface for 15 min followed by copious washing with 2 % Hellmanex (Hellma, UK) and ethanol with >18 MΩ water washes between each step. The sensor was immersed in a 1 % (*v/v*) β-mercaptoethanol (BME) in ethanol solution to passivate the gold surface (Terrettaz et al. 2002), washed with 1 % (*w/v*) SDS followed by >18 MΩ water. The running buffer for all experiments was phosphate buffered saline (PBS, 20 mM sodium phosphate pH 7.6, 137 mM NaCl and 2.7 mM KCl). The volumes and flow rates used for the assembly of the GGZctOmpA array; binding and crosslinking of antibody; and binding of antigen and secondary antibody are outlined in Supplementary Tables S2, S3 and S4 respectively with experiments repeated in triplicate.

### Quartz crystal microbalance with dissipation (QCM-D)

The QsenseE4 instrument (Q-Sense, Gothenburg, Sweden) with a peristaltic pump (Ismatec SA, Glattbrugg, Switzerland) used a flow rate of 50 μL min^−1^, a constant temperature of 24 °C and gold-coated sensor crystals (QSX-301, Q-sense). The sensor crystals were cleaned and prepared with BME in the same way as for SPR gold surfaces. The change in frequency (Δf) was measured at its fundamental frequency (5 MHz) of the quartz crystal and for the third, fifth, seventh, ninth and eleventh overtones of the fundamental frequency. Data was processed into frequency and dissipation *vs.* time with four individual experiments carried out to test for repeatability. A decrease in frequency corresponds to an increased mass on the surface of the sensor. For each overtone the change in dissipation (ΔD) was also measured. The dissipation is the proportion of energy dissipated during one cycle of the frequency oscillation and provides information on the viscoelastic properties of the materials deposited on the sensor surface. Further details can be found in the supplementary information.

### Neutron reflectometry (NR)

NR experiments were carried out on the Platypus time-of-flight neutron reflectometer (James et al. 2011; Saerbeck et al. 2012) at the 20 MW OPAL research reactor (Sydney, Australia) and on the Polref instrument (Webster et al. 2011) at the ISIS pulsed neutron source (Didcot, UK). Both reflectometers were used in polarised mode to enable magnetic contrast neutron reflectometry (Holt et al. 2009; Kirby et al. 2012). The collected data was normalised to direct beam runs collected under identical conditions. Absolutely scaled data was analysed using the MOTOFIT reflectivity analysis software (Nelson 2006). A least squares fitting routine, which selects the best fit by minimising *χ*^2^ values between model and experimental data by varying the thickness, interfacial roughness and neutron scattering length density (nSLD) of each layer, was utilised. Error analysis was carried out using a Monte Carlo resampling procedure (Heinrich et al. 2009; Holt et al. 2009) (see supplementary information).

## Results

### Assembly of the GGZctOmpA array

The assembly of the GGZctOmpA array was carried out using established methods on a gold surface passivated with BME to improve protein assembly and maintain structure (Cisneros et al. 2006; Holt et al. 2009; Keegan et al. 2005; Terrettaz et al. 2002). The GGZctOmpA was incubated on the gold surface and this was followed with an incubation of a lipid mimic of 1-mercaptoundecyl-11-triethylene glycol (thioPEG) which supports the GGZctOmpA and reduces non-specific binding in subsequent steps. Each incubation step is followed with a 1 % SDS wash to remove non-specifically bound material and a PBS wash to remove excess SDS (Supplementary Figs. S2 and S3). QCM-D showed that 1.36 ± 0.04 mg m^−2^ of GGZctOmpA remained bound on the surface after a single 20 min incubation of protein solution followed by SDS/PBS wash steps (Supplementary Fig. S3). SPR analysis of the assembly process showed that a higher surface coverage can be achieved with multiple incubation/wash steps with the gold surface saturated with GGZctOmpA at approximately 3550 RU after three incubation/wash steps (Supplementary Fig. S2). This corresponds to 3.55 mg m^−2^ of protein on the surface (assuming 1 RU = 0.001 mg m^−2^ (Stenberg et al. 1991)) and agrees with previous published data on the assembly of ZZctOmpA (Le Brun et al. 2011a). The QCM-D shows low values of ΔD and all overtones overlapping (Supplementary Fig. S3a, red trace) indicating that the monolayer is complete with little solvent content and is rigid.

**Fig. 1:**
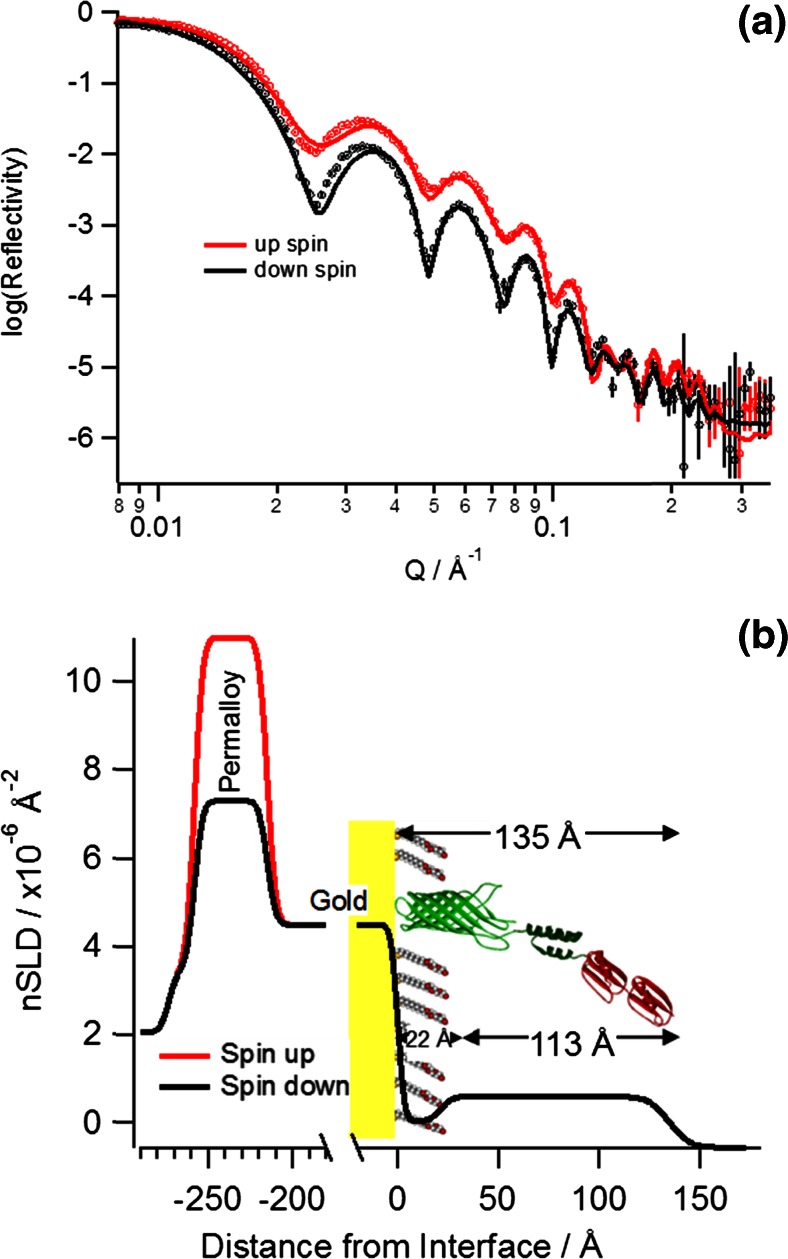
**a** Magnetic contrast neutron reflectivity (points with *error bars*) and fit (*solid lines*) of an array of deuterated GGZctOmpA surrounded with thioPEG in a H_2_O contrast. **b** The corresponding real-space nSLD profile with zero distance set as the interface between gold on the array. The gold layer has been truncated to show Permalloy layer and its different nSLD between spin up (*red*) and spin down (*black*) neutrons, and the array layers (with an illustration in the background to depict the different array layers) in detail

Neutron reflectometry provides real space structural data for surfaces and interfaces along the axis perpendicular to the lateral plane of the surface. The layer structure consisted of the silicon wafer followed by the Permalloy layer that acts as a binder between the silicon and the gold layer then finally the array which is assembled on the gold surface. Based on the results from the SPR experiments three protein incubations and SDS/buffer washes were used to assemble GGZctOmpA on gold surfaces for NR measurements. A measurement of GGZctOmpA only on the gold surface was not carried out using NR as it has been previously shown that array proteins based on the ctOmpA scaffold can adopt many orientations on the surface unless supported by a monolayer of thioPEG (Le Brun et al. 2011a). For best fitting of the NR data the array layer had to be split into two discrete layers: one layer of the thioPEG plus GGZctOmpA (plus solvent) which was 22 ± 2 Å thick and a second layer of GGZctOmpA and solvent only at 113 ± 16 Å (Fig. 1). This made a total thickness of 135 ± 18 Å for the GGZctOmpA (Fig. 1) which is consistent with similar array proteins such as ZZctOmpA at 128 Å (Le Brun et al. 2011a). The neutron scattering length density (nSLD) can be considered the neutron’s refractive index and is sensitive to the isotopic composition and physical density of each layer. By comparing theoretical nSLD values with those obtained from the fitted nSLD a volume fraction for each component can be derived. A deuterated version of the GGZctOmpA was produced (Supplementary Table S1) as the difference in scattering length between hydrogen and its stable isotope deuterium will allow a deuterated GGZctOmpA to be distinguished between hydrogenous thioPEG and IgG. Fig. 1 shows the NR data from the array in a H_2_O contrast and the 113 Å layer with a higher nSLD then that of the thioPEG containing layer and H_2_O as expected. Analysis of the nSLD values of the thioPEG plus GGZctOmpA layer shows that there is some solvent associated with the layer as there is a D_2_O/H_2_O dependent change in the nSLD and the volume fraction of solvent is 0.143 ± 0.019 (Table 1). This corresponds with the low dissipation values seen in the QCM-D trace once the array is complete (Supplementary Fig. S3a). From the nSLD of the GGZctOmpA only layer the volume fraction of the d-GGZctOmpA is 0.224 ± 0.018 and this corresponds to a surface coverage of 0.93 ± 0.20 mg m^−2^, which is comparable to the values observed for QCM-D although lower than the SPR results.

**Table 1:** The fitted properties of the completed array from neutron reflectometry

Layer	Thickness / Å	nSLD / ×10^−6^ Å^−2^	Volume fraction	Roughness / Å
Permalloy	43 ± 1	7.36 ± 0.10 (down)	–	4
		11.06 ± 0.13 (up)		
Gold	231 ± 3	4.50^a^	–	4
thioPEG + d-GGZctOmpA	22 ± 2	1.19 ± 0.16 (D_2_O)	0.633 ± 0.085 (thioPEG only)	5
		0.20 ± 0.04 (H_2_O)		
d-GGZctOmpA only	113 ± 16^b^	0.64 ± 0.05 (H_2_O)	0.224 ± 0.018	5
	18 ± 3^c^			
mAb245 only	135 ± 11	6.33 ± 0.05 (D_2_O)	0.067 ± 0.007	5
		−0.45 ± 0.09 (H_2_O)		
mAb245 + rNP	201 ± 22	6.19 ± 0.04 (D_2_O)	0.095 ± 0.015 (rNP only)	5
		0.40 ± 0.12 (H_2_O)		
Subphase	N/A	6.35 (D_2_O)^a^	–	18
		−0.56 (H_2_O)^a^		

^a^nSLD values were fixed at the values quoted in fits

^b^thickness value before antibody addition

^c^thickness when mAb245 is bound

### Antibody and antigen binding

**Fig. 2:**
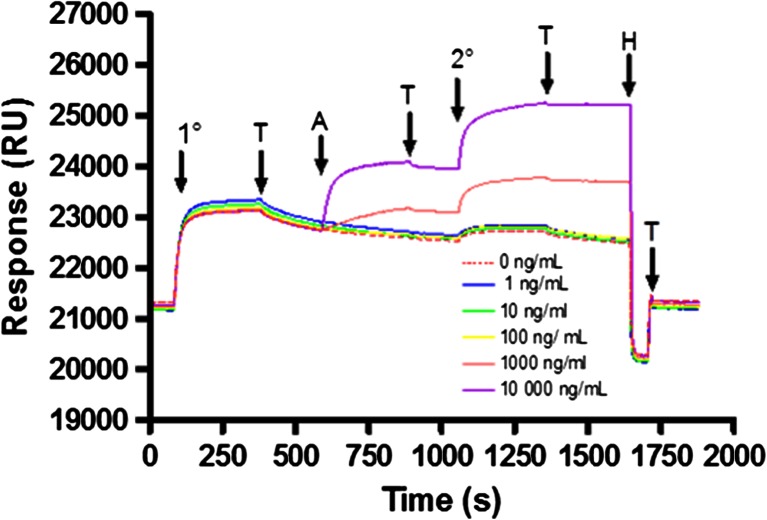
The binding of antibody and antigen to arrays of GGZctOmpA measured by SPR. 30 μg mL^−1^ mAb245 was bound to an array of GGZctOmpA, then rNP at a concentration range of 0 to 10 000 ng mL^−1^ was bound. Secondary antibody, mAb108 (30 μg mL^−1^), was the final binding event before regeneration with 100 mM HCl. 1° - mAb245 injection; T – buffer wash; A – rNP injection, 2° - mAb108 injection, H – regeneration with 100 mM HCl

**Fig. 3:**
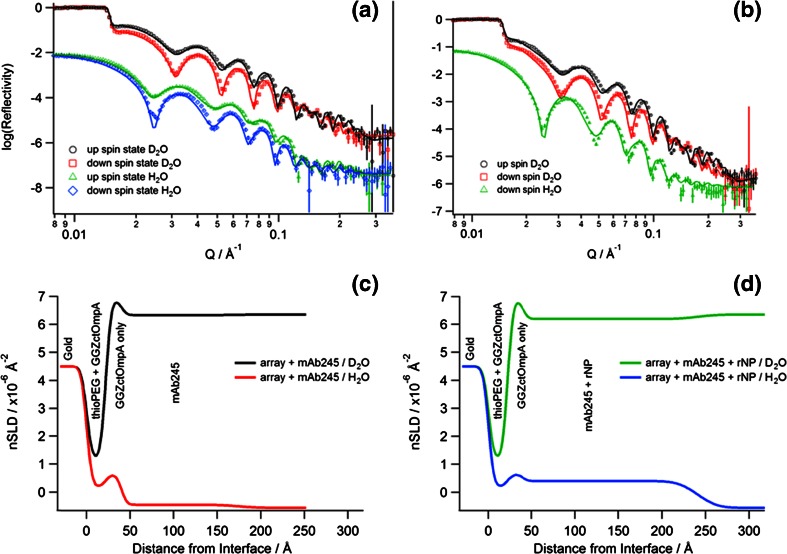
Reflectivity data (*symbols*) and fit (*solid lines*) of an array of d-GGZctOmpA filled with thioPEG and with **a** mAb245 bound only to the array and **b** is the reflectivity data of the array with mAb245 and rNP bound. Both data have the same symbols and shows the up spin states (○ and □) and down spin states (□ and ◇) as well as the D_2_O (○ and □) and H_2_O (△ and ◇) contrasts (spin up H_2_O absent in **b**). The data between the D_2_O and H_2_O contrasts is offset for clarity. **c** The corresponding real-space nSLD profiles of the array with mAb245 bound from the gold interface and d) is the corresponding real-space nSLD profile of the array from the gold interface with mAb245 and rNP bound

Binding of mouse monoclonal anti-influenza type A IgG2b (mAb245) to the array was measured by SPR and QCM-D (Supplemental Figs. S4 and S5). The binding of mAb245 over a range of concentrations was investigated using SPR and showed saturation of the array at high concentrations (Supplemental Fig. S4). Figure 2 and Supplemental Fig. S4 demonstrate that the mAb remains tightly bound to the array after injection, evidenced by the minimal dissociation observed. A high surface coverage of GGZctOmpA was used to best reflect a real device and gain the maximum amount of mAb binding, therefore the k_on_ and k_off_ (and hence the K_d_) cannot be accurately determined between individual IgG and GGZctOmpA molecules due to mass transport and avidity affects. QCM-D experiments were carried out to complement the SPR data and found that typically 1.50 ± 0.07 mg m^−2^ of mAb245 bound to the array (Supplemental Fig. S5). The QCM-D data further confirmed slow dissociation from the array (Supplemental Fig. S5, 25 to 40 min) which is an improvement compared to when the IgG capture protein is the Z domain of protein A (Le Brun et al. 2011a). As shown by the increase in dissipation (Supplemental Fig. S5, red trace), bound mAb245 is non-rigid and no longer obeys the Sauerbrey equation. The array was measured by NR with mAb bound, using both spin up and spin down neutrons as well as using a D_2_O and H_2_O contrast (Fig. 3a). The real-space nSLD profile showed that the IgG layer has to be modelled as a single layer with a thickness of 135 ± 11 Å (Table 1 and Fig. 3c), consistent with an IgG molecule that is in the ‘up right’ position with the variable domain facing towards the bulk solvent. The NR shows that there is a high water content in the mAb245 layer with only a 0.067 ± 0.007 volume fraction of protein. This will be due to the low initial surface coverage of the GGZctOmpA and explains the high dissipation seen the QCM-D data. Between the 135 Å IgG layer and the 22 Å thioPEG layer there was an 18 Å layer of high nSLD which corresponds to the deuterated GGZctOmpA only (Fig. 3c). This shows that the IgG does not sit on the protein resistant thioPEG layer.

**Fig. 4:**
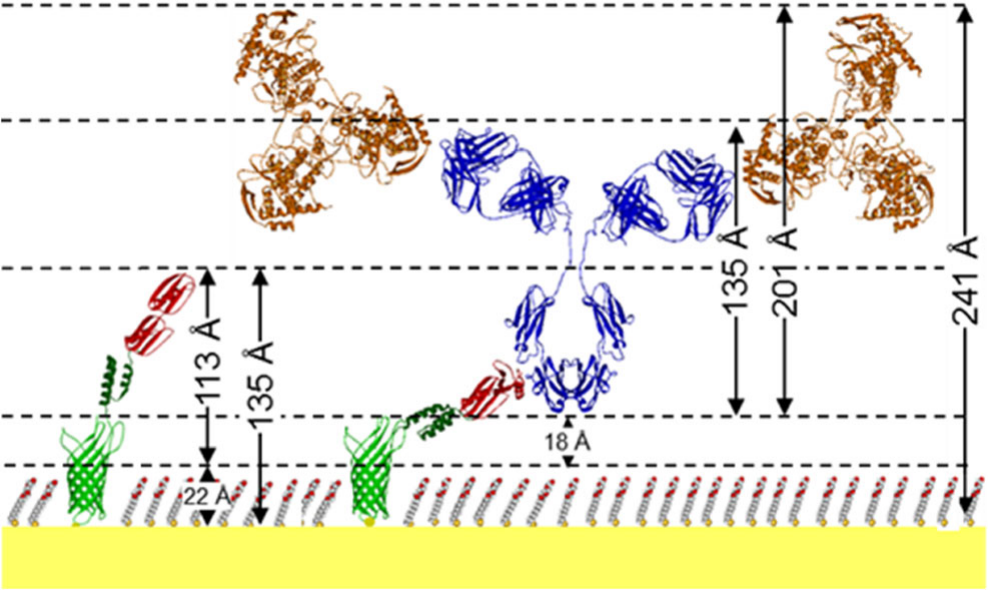
The dimensions of each component of a GGZctOmpA array before and after IgG and rNP binding. ctOmpA is shown in light green and is bound to the gold surface and is surrounded by thioPEG (the molecular packing is not represented accurately) with a protein A Z domain linker (*dark green*) and a tandem pair of B domains from protein G (*red*) to which there is an IgG (*blue*) bound at the Fc region. Bound to the IgG Fab regions there is NP (*orange*) to which a secondary IgG can be bound (not shown)

The antigen, rNP, binds to the array through the antigen binding domains of mAb245 and both SPR and QCM-D confirm that rNP binds to GGZctOmpA-immobilised mAb245 (Fig. 2 and Supplemental Fig. S5). The rNP binding was tested over a concentration range of 1 to 10 000 ng mL^−1^ by SPR (Fig. 2). Bound rNP was measured under the same conditions as for bound mAb245 using NR (Fig 3b). The measured NR data with rNP bound show small changes in the shape of the fringes when compared to mAb245 only, suggesting that rNP has bound but there is no great structural change. The modelling of the NR data showed that when bound to the array, rNP antibody layer still had to be modelled as one discrete layer (Fig. 3d) as separating the antibody layer into two layers of antibody and antigen resulted in poorer fits. On binding of rNP to the antibody layer the overall thickness of the layer increased to 201 ± 22 Å, strongly indicating that antigen is bound (Fig. 3d and Table 1). There is little change in the nSLD of the antibody layer after rNP addition and this is unsurprising due to the low surface coverage of mAb245. However, the Monte Carlo resampling for the nSLD parameter shows a clear difference in nSLD before and after rNP addition (Supplementary Fig. S6) confirming rNP binding in Fig. 3c. Figure 4 shows the dimensions for each component in the GGZctOmpA array plus rNP and shows that the total thickness for the array is 241 ± 27 Å.

The secondary antibody used was a mouse monoclonal anti-influenza virus type A IgG1 (mAb108) that was raised against a different epitope of the NP protein. Binding mAb108 enhances the signal from bound rNP as shown by SPR (Fig. 2). Although we completely saturate the available protein G domains with IgG there is some dissociation of the primary IgG (mAb245) after injection which could result in trace amounts of free protein G domains. The secondary antibody (which is also a mouse monoclonal IgG) can bind to any free protein G molecules which would result in some background signal in the 0 ng/mL rNP case. The mass of secondary antibody that bound to the array was 3.69 ± 0.04 mg m^−2^ as determined by QCM-D (Supplemental Fig. S5). This is a binding ratio of mAb108 to rNP of 1:0.65 (expected 1:1). The large increase in QCM-D dissipation shows that mAb108 is non-rigid when bound (Supplemental Fig. S5a, red trace).

Specificity of rNP binding and the level of non-specific binding was tested with a series of control SPR experiments. The first experiment was binding mAb245 and rNP to an array of ctOmpA where the tandem pair of protein G domains is absent and therefore cannot specifically capture IgG. Supplementary Fig. S7 shows that neither mAb245 nor rNP can bind to an array of ctOmpA. A second experiment was carried out where rNP was bound to an GGZctOmpA with a IgG bound which is not specific for rNP. Supplemental Fig. S8 shows the binding of rNP to mouse monoclonal anti-FLAG M2 IgG1 (which is specific for the FLAG epitope) immobilised to an array of GGZctOmpA. Supplemental Fig. S8b is a comparison of the relative response of rNP binding to mAb245 and anti-FLAG M2 immobilised to GGZctOmpA with time zero set as the point of 1 μg mL^−1^ rNP injection. The trace shows that rNP does not bind to the anti-FLAG M2 IgG1 but does bind to mAb245.

### Cross-linking assays

**Fig. 5:**
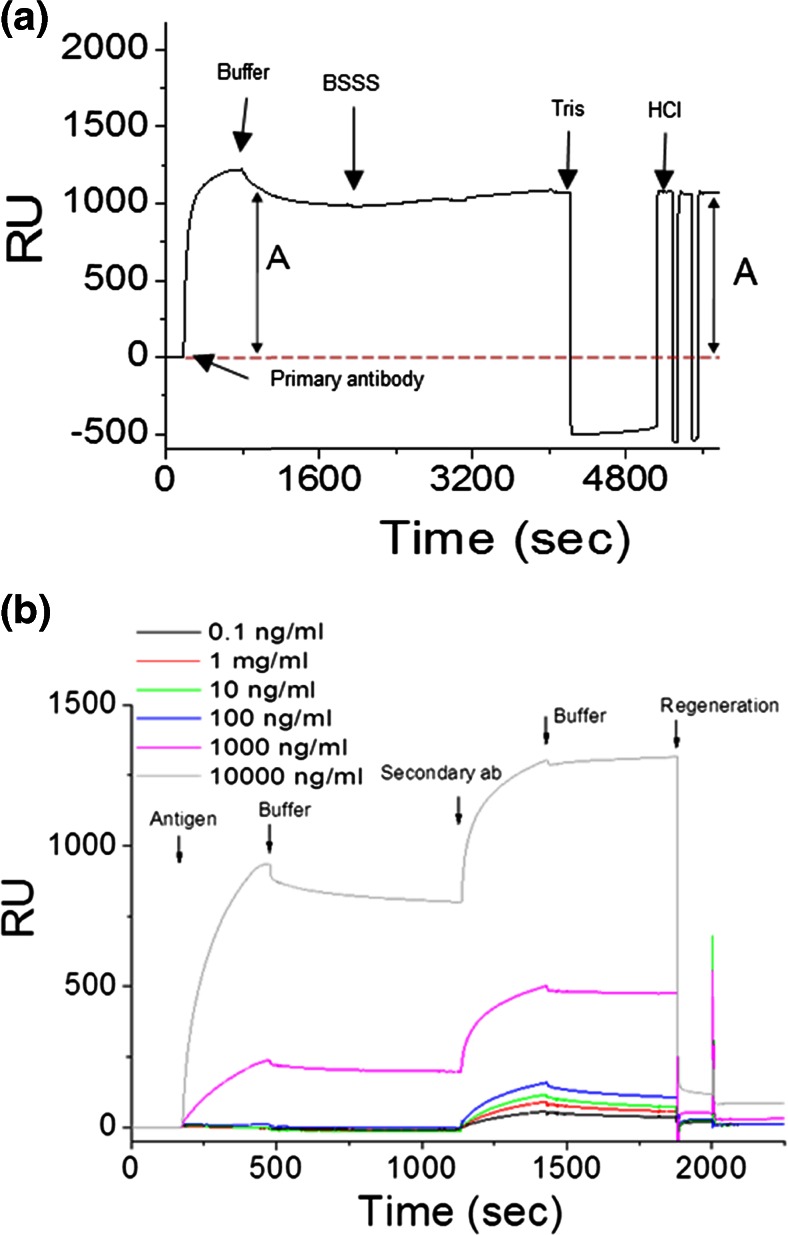
**a** Cross-linking IgG to an array of GGZctOmpA with BSSS. 30 μg mL^−1^ of mAb245 was incubated on the surface for 300 s at a flow rate of 5 μL min^−1^ this was followed by wash with PBS buffer. An incubation of 0.5 mM BSSS for 900 s was carried out to initialise cross-linking with the reaction terminated with 1 M Tris–HCl pH 6.8 followed by a double wash of 100 mM HCl. The before and after response to the crosslinking was assessed in the areas marked ‘A’ **b** Binding of rNP (over a concentration range of 0.1 to 10 000 ng mL^−1^) to mAb245 that has been cross-linked to an array of mAb245 with BSSS cross-linker followed by binding of mAb108 (30 μg mL^−1^) at a flow rate of 5 μL min^−1^. Between each concentration the array was regenerated with a wash of 100 mM HCl. Data was subtracted from a data set of 0 ng mL^−1^ antigen

To further reduce the dissociation of IgG from GGZctOmpA arrays the IgG was chemically cross-linked to the protein G domains of GGZctOmpA in separate experiments by two different cross-linkers. The first was Bis(sulfosuccinimidyl) suberate (BSSS) which is an *N*-hydroxysulfosuccinimidyl (NHS)-ester homobifunctional water soluble cross-linker with an eight carbon spacer arm of 11.4 Å. The second linker was Bis(succinimidyl) penta(ethylene glycol) (BS(PEG)_5_), which is a linker with a pentaethylene glycol spacer of 21.7 Å. Both cross-linkers are amine – to – amine cross-linkers that are ideal for cross-linking IgG which have many surface exposed lysine residues. The cross-linking reagent at a final concentration of 0.5 mM in PBS buffer was incubated with the preformed IgG array and the reaction was terminated with 1.0 M Tris–HCl pH 6.8 (Fig. 5a and Supplemental Fig. S9). Non-cross-linked antibody was removed by washing with 100 mM HCl followed by a PBS wash. The effectiveness of the cross-linking was assessed by comparing the amount of mAb245 bound to the array before the addition of the cross-linking agent to the amount bound after the HCl and PBS wash steps (the areas marked with the letter ‘A’ in Fig. 5a). Both cross-linking agents were found to cross-link with approximately 98 ± 1 % efficiency. The effect of the cross-linking on the array’s ability to bind rNP and mAb108 was tested and mAb245 cross-linked to an array of GGZctOmpA retained the capacity to bind rNP. By comparing Figs. 2 and 5 cross-linked mAb245 binds rNP to the same extent as non-cross-linked mAb245 across all bulk rNP concentrations. Furthermore, the array can be regenerated, by washing with 100 mM HCl to remove the rNP, leaving the cross-linked mAb245 in place (Fig. 5). This allows for multiple cycles of antigen binding and array regeneration without the need to replenish the array with primary IgG on each new addition of antigen. Secondary antibody (mAb108) binding to antigen captured by cross-linked mAb245 was also demonstrated with SPR (Fig. 5b).

## Discussion

The analytical methods provide a comprehensive picture of the processes occurring during self-assembly and the combination of SPR, QCM-D and NR provides both overlap and complementarity. Protein G B-domains were successfully fused to the ctOmpA scaffold with a protein A Z-domain as a linker molecule. The structure of GGZctOmpA could be checked in solution using circular dichroism spectroscopy (Supplementary Fig. S1) before forming self-assembled monolayers on gold surfaces (Fig. 1 and Supplementary Figs. S2 and S3) as shown for other β-barrelled scaffold proteins (Le Brun et al. 2011a, b; Shah et al. 2007; Terrettaz et al. 2002). Specific deuteration of just GGZctOmpA allowed for this array protein to be distinguished from the hydrogenous thioPEG and IgG and clearly highlights the 18 Å layer between the thioPEG / array protein layer and the IgG layer (Fig. 3c). Although similar layers have been observed previously (Le Brun et al. 2008, 2011a), all components were hydrogenous so that the gap between the IgG and the protein resistant thioPEG layer was previously unseen.

Protein G has previously been bound to surfaces and the linkers used can be split into two categories resulting in either oriented immobilisation or random immobilisation (Trilling et al. 2013). This GGZctOmpA array clearly binds IgG in an oriented manner with the neutron reflectivity data showing that the IgG layer is 135 Å thick. Considering the dimensions of IgG (143 × 77 × 40 Å^3^ (Silverton et al. 1977)), this would indicate an orientation which is optimal for antigen binding where the variable, antigen-binding domains are facing away from the surface (Figs. 3c and 4). Although protein G can also bind the antigen-binding Fab fragments of IgG (Tashiro and Montelione 1995), isothermal titration calorimetry has shown that the K_a_ of protein G binding to Fab fragments was two orders of magnitude lower than binding to Fc fragments or whole IgG, and that binding to the Fc domain dominated (Lund et al. 2011).

Strong binding with little dissociation between the GGZctOmpA array and mouse monoclonal IgG2b was demonstrated (Fig. 2). Arrays of ZZctOmpA, which capture IgG using the Z domain of *Staphylococcal aureus* protein A, bound mouse monoclonal IgG2a with a K_d_ of 0.778 nM (Le Brun et al. 2011a), which also indicates strong binding. However, the subclass and species of origin of the IgG has to be taken into account. Unlike mouse monoclonal IgG2a, mouse monoclonal IgG2b and IgG1 bind to protein A poorly (Björck and Kronvall 1984), whereas protein G binds all mouse monoclonal IgG subclasses (Björck and Kronvall 1984; Eliasson et al. 1988). Therefore the advantage of using protein G over protein Z as the IgG binding domain is the ability of protein G to bind a much wider range of IgG classes than protein A (Björck and Kronvall 1984). Eliasson et al. (1988) showed that creating genetic fusions of the IgG-binding domains of protein A and protein G can have additive effects. In this work a Z domain from protein A was used as a linker between ctOmpA and protein G so the possibility of some additive effect in this array exists.

Previous work has shown that a greater antigen binding can be achieved through oriented immobilisation of IgG to surfaces (Bergström and Mandenius 2011; Kausaite-Minkstimiene et al. 2010; Song et al. 2011) and this work demonstrated complete saturation of the antigen binding sites on the IgG (Fig. 2). Although complete saturation was achieved, a discrete antigen layer could not be resolved in the NR data; rather an overall increase in the nSLD and thickness of the antibody layer was observed (Fig. 3d and Supplementary Fig. S6). This indicates that the rNP, can adopt multiple IgG-bound orientations allowed by the flexible hinge region between Fc and Fab domains which orientates Fab in different arrangements relative to the position of Fc without affecting the overall shape of the IgG (Abe et al. 2010; Harris et al. 1997; Saphire et al. 2002). This is reinforced by the increased dissipation in the QCM-D traces suggesting flexibility in the bound IgG structure (Supplementary Fig. S5). In Fig. 4 we only show one IgG arrangement for clarity; however the Figure does indicate that IgG fully occupied with rNP would occupy a large molecular area and this could account for the low volume fractions observed. SPR and QCM-D data shows that the IgG to antigen ratio of mAb245 to rNP binding is higher than that observed previously with an array of ZZctOmpA using rabbit polyclonal anti-HSA IgG and human serum albumin as antigen where a 25 % occupancy of the antigen binding sites was achieved (Le Brun et al. 2011a).

The engineering of the array *via* the chemical cross-linking of IgG to the protein G moieties prevents IgG dissociation while retaining the detection capabilities of the original array (Fig. 5). In addition crosslinking the IgG to protein G stabilises the array enabling regeneration without having to replenish primary antibody *via* a simple low pH solution wash. A number of approaches have been used to cross-link protein G or protein A to IgG for enhanced stability which include using cyanamide (Bereli et al. 2011), dimethyl pimelimidate (Bergström and Mandenius 2011), and bis(sulfosuccinimidyl) linkers(Song et al. 2011). In this work bis(sulfosuccinimdyl) linkers were used due to their mild conditions for cross-linking and ease of use. The functional ends of the cross-linkers were separated by different spaced cross-linkers with each successfully cross-linking the IgG and protein G. Previous cross-linking studies, have found that the orientation of the IgG is more important than the cross-linking reagent (Kausaite-Minkstimiene et al. 2010) and this work supports this.

Previous work creating membrane protein – immunoglobulin-binding domain chimeras have only been tested against IgG that have been raised against model proteins such as albumin (Le Brun et al. 2011a, b). By creating the GGZctOmpA molecule a wider range of monoclonal IgG subtypes can be bound which increases the prospect for a larger range of epitopes from ‘real world’ analytes to be detected. In this work the molecule that was chosen for proof-of-concept was recombinant influenza A nucleoprotein which is currently the basis of a number of point-of-use diagnostic tests for influenza (Hatchette 2009; Peters et al. 2013).

## Conclusions

This work has revealed how nanoscale components (engineered protein, alkane thiol and IgG) can self-assemble into an array onto surfaces for potential microscale devices. The antigen used for the proof of concept was nucleoprotein from influenza, creating a label-free platform that is amenable to both SPR and QCM-D techniques and sensitive to the presence of flu antigens. Crosslinking the IgG to the array enables the benefits of non-covalent self-assembly to be used in a stable covalent structure. Probing the interfacial structure of the array by neutron reflectometry using both conventional isotope contrasts and magnetic contrasts showed a structured array with optimal IgG orientation for antigen capture.

## Electronic supplementary material

ESM 1 (DOCX 266 kb)

## Abbreviations

BS(PEG)_5_: Bis(succinimidyl) penta(ethylene glycol)
BSSS: Bis(sulfosuccinimidyl) suberate
ctOmpA: Circularly permuted transmembrane domain of *E. coli* outer membrane protein A
GGZctOmpA: N-terminal tandem pair of B domains from protein G with a C-terminal ctOmpA scaffold
IgG: Immunoglobulin G
mAb245: Mouse monoclonal IgG raised against rNP
mAb108: Mouse monoclonal IgG raised against rNP (different epitope to mAb245)
NP: Native nucleoprotein from influenza
NR: Neutron reflectometry
nSLD: Neutron scattering length density
PBS: Phosphate buffered saline
QCM-D: Quartz-crystal microbalance with dissipation monitoring
rNP: Recombinant nucleoprotein
SPR: Surface plasmon resonance
thioPEG: 1-mercaptoundec-11-yltriethylene glycol

## References

[CR1] Abe Y, Gor J, Bracewell DG, Perkins SJ, Dalby PA (2010). Biochem. J..

[CR2] Akerström B, Brodin T, Reis K, Björck L (1985). J. Immunol..

[CR3] Amano Y, Cheng Q (2005). Anal. Bioanal. Chem..

[CR4] Bao Y, Bolotov P, Dernovoy D, Kiryutin B, Zaslavsky L, Tatusova T, Ostell J, Lipman D (2008). J. Virol..

[CR5] Bereli N, Şener G, Yavuz H, Denizli A (2011). Mater. Sci. Eng. C.

[CR6] Bergström G, Mandenius C-F (2011). Sens. Actuators B.

[CR7] Björck L, Kronvall G (1984). J. Immunol..

[CR8] Cisneros DA, Muller DJ, Daud SM, Lakey JH (2006). Angew. Chem. Int. Ed..

[CR9] Coloma R, Valpuesta JM, Arranz R, Carrascosa JL, Ortín J, Martín-Benito J (2009). PLoS Pathog..

[CR10] Cornell BA, Braach-Maksvytis VLB, King LG, Osman PDJ, Raguse B, Wieczorek L, Pace RJ (1997). Nature.

[CR11] Cowsill BJ, Zhao X, Waigh TA, Eapen S, Davies R, Laux V, Forsyth VT, Haertlein M, Lu JR (2014). Langmuir.

[CR12] Dubois LH, Nuzzo RG (1992). Annu. Rev. Phys. Chem..

[CR13] Eliasson M, Olsson A, Palmcrantz E, Wiberg K, Inganäs M, Guss B, Lindberg M, Uhlén M (1988). J. Biol. Chem..

[CR14] Farris L, Wu N, Wang W, Clarizia L-J, Wang X, McDonald M (2010). Anal. Bioanal. Chem..

[CR15] Fletcher JM, Harniman RL, Barnes FRH, Boyle AL, Collins A, Mantell J, Sharp TH, Antognozzi M, Booth PJ, Linden N, Miles MJ, Sessions RB, Verkade P, Woolfson DN (2013). Science.

[CR16] Gallagher T, Alexander P, Bryan P, Gilliland GL (1994). Biochemistry.

[CR17] Gao D, McBean N, Schultz JS, Yan Y, Mulchandani A, Chen WJ (2006). J. Am. Chem. Soc..

[CR18] Ha TH, Jung SO, Lee JM, Lee KY, Lee Y, Park JS, Chung BH (2006). Anal. Chem..

[CR19] Harris LJ, Larson SB, Hasel KW, McPherson A (1997). Biochemistry.

[CR20] Hatchette TF (2009). Can. J. Public Health.

[CR21] Heinrich F, Ng T, Vanderah DJ, Shekhar P, Mihailescu M, Nanda H, Losche M (2009). Langmuir.

[CR22] Ho J-AA, Hsu W-L, Liao W-C, Chiu J-K, Chen M-L, Chang H-C, Li C-C (2010). Biosens. Bioelectron..

[CR23] Holt SA, Le Brun AP, Majkrzak CF, McGillivray DJ, Heinrich F, Loesche M, Lakey JH (2009). Soft Matter.

[CR24] Huy TQ, Hanh NTH, Van Chung P, Anh DD, Nga PT, Tuan MA (2011). Appl. Surf. Sci..

[CR25] James M, Nelson A, Holt SA, Saerbeck T, Hamilton WA, Klose F (2011). Nucl. Instrum. Meth. A.

[CR26] Jung S-H, Son H-Y, Yuk JS, Jung J-W, Kim KH, Lee C-H, Hwang H, Ha K-S (2006). Coll. Surf. B: Biointerface.

[CR27] Jyoung J-Y, Hong S, Lee W, Choi J-W (2006). Biosens. Bioelectron..

[CR28] Kausaite-Minkstimiene A, Ramanaviciene A, Kirlyte J, Ramanavicius A (2010). Anal. Chem..

[CR29] Keegan N, Wright NG, Lakey JH (2005). Angew. Chem. Int. Ed..

[CR30] Kirby BJ, Kienzle PA, Maranville BB, Berk NF, Krycka J, Heinrich F, Majkrzak CF (2012). Curr. Opin. Colloid Interface Sci..

[CR31] Kogai T, Yoshimura N, Mori T, Yatsuda H (2010). Jpn. J. Appl. Phys..

[CR32] Le Brun AP, Holt SA, Shah DS, Majkrzak CF, Lakey JH (2008). Eur. Biophys. J..

[CR33] Le Brun AP, Holt SA, Shah DSH, Majkrzak CF, Lakey JH (2011). Biomaterials.

[CR34] Le Brun AP, Shah DSH, Athey D, Holt SA, Lakey JH (2011). Int. J. Mol. Sci..

[CR35] Liu X, Wang X, Zhang J, Feng H, Liu X, Wong DKY (2012). Biosens. Bioelectron..

[CR36] Love JC, Estroff LA, Kriebel JK, Nuzzo RG, Whitesides GM (2005). Chem. Rev..

[CR37] Lund LN, Christensen T, Toone E, Houen G, Staby A, St Hilaire PM (2011). J. Mol. Recognit..

[CR38] Makaraviciute A, Ramanaviciene A (2013). Biosens. Bioelectron..

[CR39] Nelson A (2006). J. Appl. Crystallogr..

[CR40] Oh B-K, Lee W, Kim Y-K, Lee W-H, Choi J-W (2004). J. Biotechnol..

[CR41] Peters TR, Blakeney E, Vannoy L, Poehling PA (2013). Diagn. Microbiol. Infect. Dis..

[CR42] Prime KL, Whitesides GM (1993). J. Am. Chem. Soc..

[CR43] Roth MJ, Maresh EM, Plymire DA, Zhang J, Corbett JR, Robbins R, Patrie SM (2013). ACS Appl. Mater. Interfaces.

[CR44] Ruigrok RWH, Crépin T, Hart DJ, Cusack S (2010). Curr. Opin. Struct. Biol..

[CR45] Saerbeck T, Klose F, Le Brun AP, Fuzi J, Brule A, Nelson A, Holt SA, James M (2012). Rev. Sci. Instrum..

[CR46] Sambrook J, Russell DW (2001). Molecular cloning.

[CR47] Saphire EO, Stanfield RL, Crispin MDM, Parren P, Rudd PM, Dwek RA, Burton DR, Wilson IA (2002). J. Mol. Biol..

[CR48] Sauer-Eriksson AE, Kleywegt GJ, Uhlén M, Jones TA (1995). Structure.

[CR49] Shah DS, Thomas MB, Phillips S, Cisneros DA, Le Brun AP, Holt SA, Lakey JH (2007). Biochem. Soc. Trans..

[CR50] Shen G, Cai C, Wang K, Lu J (2011). Anal. Biochem..

[CR51] Silverton EW, Navia MA, Davies DR (1977). Proc. Natl. Acad. Sci. U. S. A..

[CR52] Skottrup PD, Nicolaisen M, Justesen AF (2008). Biosens. Bioelectron..

[CR53] Song HY, Zhou X, Hobley J, Su X (2011). Langmuir.

[CR54] Stenberg E, Persson B, Roos H, Urbaniczky C (1991). J. Colloid Interface Sci..

[CR55] Tashiro M, Montelione GT (1995). Curr. Opin. Struct. Biol..

[CR56] Terrettaz S, Ulrich WP, Vogel H, Hong Q, Dover LG, Lakey JH (2002). Protein Sci..

[CR57] Trilling AK, Beekwilder J, Zuilhof H (2013). Analyst.

[CR58] Webster JRP, Langridge S, Dalgliesh RM, Charlton TR (2011). Eur. Phys. J. Plus.

[CR59] Xu H, Lu JR, Williams DE (2006). J. Phys. Chem. B.

[CR60] Xu H, Zhao X, Grant C, Lu JR, Williams DE, Penfold J (2006). Langmuir.

[CR61] Xu H, Zhao X, Lu JR, Williams DE (2007). Biomacromolecules.

[CR62] Ye Q, Krug RM, Tao YJ (2006). Nature.

[CR63] Yu H, Qiu X, Nunes SP, Peinemann K-V (2014). Angew. Chem. Int. Ed..

