# Supplementary material for: Engineered self-assembling monolayers for label free detection of influenza nucleoprotein
Source: Biomed Microdevices. 2015 Apr 11;17(3):49. doi: 10.1007/s10544-015-9951-z (PMC4392172; doi:10.1007/s10544-015-9951-z)
Supplement: Supplementary file 1 — (DOCX 266 kb) [file 10544_2015_9951_MOESM1_ESM.docx]

**ELECTRONIC SUPPLEMENTARY MATERIAL**

**Engineered Self-Assembling Monolayers for Label Free Detection of Influenza Nucleoprotein**

Anton P. Le Brun, Andrei Soliakov, Deepan S. H. Shah, Stephen A. Holt,

Alison McGill and Jeremy H. Lakey

**Supplementary Materials and Methods**

**GGZctOmpA expression and purification**

GGZctOmpA was expressed as insoluble inclusion bodies in 1L flask cultures in LB broth (+ 100 µg/mL ampicillin) induced with 1 mM IPTG for 6h at 37°C. The cells were harvested by centrifugation (3000x*g* 10 min) and lysed in 25 mL BugBuste srolution (Novagen) containing lysozyme at 10 µg/mL and Benzonase Nuclease (Novagen) at 0.25 units/mL. The cells were stored overnight at -20°C. The lysate was centrifuged at 12000x*g* to pellet the inclusion bodies. These were washed 3x with 25 mL of 1/10 diluted BugBuster solution in autoclaved deionised water. The final pellet was dissolved in Binding Buffer (20 mM Sodium phosphate pH7.4, 8 M Urea, 0.5M NaCl, 20 mM imidazole). After centrifugation and filtration to remove undissolved particulates, the solubilised inclusion body solution was loaded onto a 1 mL HisTrap HP column (GE) using an ÄKTA Prime chromatography system (GE), the column was washed with binding buffer and then the GGZctOmpA protein eluted in Elution Buffer (Binding buffer containing 250 mM imidazole). The peak fractions were collected and concentrated in a Vivaspin centrifugal concentrator with 30K molecular weight cut-off. The concentrate was passed through a PD10 desalting column (GE) equilibrated with ion exchange buffer A (IEX-A: 20 mM Ethanolamine, 8M urea, pH adjusted to 10 with acetic acid) to de-salt the preparation. The protein was eluted from the PD10 column with IEX-A. This eluent was loaded onto a pre-equilibrated 1 mL Hi Trap QFF anion exchange column (GE), washed with 10 volumes of IEX A and eluted with a NaCl gradient from 0-250 mM in IEX-A. The peak fractions were collected and analysed by SDS PAGE. Fractions containing the GGZctOmpA (~44kDa band) protein at >95% homogeneity were pooled and concentrated to sub millilitre concentration on Vivaspin centrifugal concentrator with 30K molecular weight cut-off. The protein was refolded by dilution into ROG-10 buffer (50 mM ethanolamine, 0.1 mM EDTA, 1% w/v octylglucoside, 1 mM DTT) and incubation at 37°C for 48 h. Refolding was confirmed by band-shift assay and circular dichroism spectroscopy.

**Recombinant influenza A NP expression and purification**

In order to attain soluble expression, rNP was expressed in *E. coli* BL21-AI strain (Invitrogen) in which the T7 polymerase is under the control of the arabinose inducible *araBAD* promoter. BL21-AI cells containing the pOrla108 expression plasmid were grown to an OD_600_ of ~1 in LB broth with 100 µg/mL ampicillin and then induced with 0.2% final concentration of L-arabinose. The cells were grown at 37°C for 3.5 h and then at 18°C overnight. The cells were harvested by centrifugation (3000x*g* 10 min); the pellet was resuspended in lysis buffer (5 mM imidazole, 0.5 M NaCl, 50 mM sodium phosphate buffer pH7.3) and sonicated for 30 min with a 10s ON, 30s OFF cycles at power level 6.5 (sonication time of 7.5 min). The lysate was cleared by centrifugation at 20000x*g* for 25 min at 4°C. The supernatant was diluted 1 in 2 with lysis buffer and loaded onto a gravity column containing a 10 mL bed volume of Ni-NTA resin pre-equilibrated with lysis buffer. The column was washed with 200 mL of wash buffer (20 mM imidazole, 0.5 M NaCl, 50 mM sodium phosphate at pH7.3). The bound protein was eluted with wash buffer containing 0.3M imidazole. The eluent was concentrated, diluted 1 in 10 into 50 mM sodium phosphate buffer at pH7.3 and loaded onto a pre equilibrated 5 mL HiTrap SPFF cation exchange column (GE) using an ÄKTA FPLC chromatography system. After washing with 50 mL phosphate buffer pH7.3, the bound proteins were eluted with a NaCl gradient from 0-500 nM in 50 mM Phosphate buffer pH7.3. Peak fraction was analysed by SDS PAGE to ensure >95% homogeneity of rNP (~57 kDa). The binding of anti-Influenza A NP mAbs InA245 and InA108 (HyTest) was confirmed by Western blotting (data not shown).

**Circular Dichroism (CD)**

Protein concentrations were determined by absorbance at 280 nm prior CD measurements. CD spectroscopy was carried out using 0.02 cm pathlength demountable cuvettes in a Jasco J-810 spectropolarimeter. Scans were taken from 250 nm to 185 nm ten times and averaged. A buffer blank was subtracted and the data expressed as Δε with units of M^-1^cm^-1^.

**Quartz Crystal Microbalance with Dissipation (QCM-D)**

The QsenseE4 instrument (Q-Sense, Gothenburg, Sweden) with a peristaltic pump (Ismatec SA, Glattbrugg, Switzerland) used a flow rate of 50 μL min^-1^, a constant temperature of 24 ˚C and gold-coated sensor crystals (QSX-301, Q-sense). The sensor crystals were cleaned and prepared with BME in the same way as for SPR gold surfaces. The change in frequency (Δf) was measured at its fundamental frequency (5 MHz) of the quartz crystal and for the third, fifth, seventh, ninth and eleventh overtones of the fundamental frequency (corresponding to 15, 25, 35, 45 and 55 MHz respectively). Data was processed into frequency and dissipation vs. time with four individual experiments carried out to test for repeatability. A decrease in frequency corresponds to an increased mass on the surface of the sensor ([Cooper and Singleton 2007](#_ENREF_1)). For rigid films with little water content that have minimal changes in dissipation (<1), the Sauerbrey equation can be used to relate mass (Δm) and frequency (Δf):

$$\Delta m=-\frac{\Delta f\rho_{q}v_{q}}{2\sqrt{F}n}$$

where *ρ_q_* is the density of quartz (2648 kg m^-3^), *v_q_* is the speed of sound through quartz (3340 m s^-1^), *F* is the fundamental frequency (5 MHz) and *n* is the overtone number (n = 3, 5, 7, 9 or 11). For each overtone the change in dissipation (ΔD) was also measured. The dissipation is the proportion of energy dissipated during one cycle of the frequency oscillation and provides information on the viscoelastic properties of the materials deposited on the sensor surface. For films that are non-rigid and have a dissipation which is large (>1) the Sauerbrey equation is no longer valid. In this case the data was fitted using the Voigt model ([Rodahl et al. 1997](#_ENREF_9)) within instrument software. A least-squares fitting routine was used and the data was fitted with a model that incorporates thickness, viscosity and shear modulus parameters.

**Neutron Reflectometry (NR) experiments and data analysis**

NR experiments were performed on a silicon (111) wafer of 10 cm diameter and 3 mm thickness which were coated by ion beam deposition with a ~ 50 Å layer of Permalloy and a ~ 230 Å layer of gold by INSEC (Portugal). The wafer was placed into an aluminium sample cell with silicon backing and fronting wafers. Between the gold surface and the fronting wafer was a polyester O-ring, which leaves a 100 μm deep reservoir. The fronting plate had inlet and outlet tubes to allow for sample injection and solvent exchange.

NR data was measured using the Platypus time-of-flight neutron reflectometer ([James et al. 2006](#_ENREF_4); [James et al. 2011](#_ENREF_5)) in polarised mode ([Saerbeck et al. 2012](#_ENREF_10)) on a cold neutron spectrum at the OPAL 20 MW research reactor (Sydney, Australia). Neutron pluses of 33 Hz were generated using a disc chopper system (EADS Astrium, Germany) set to a medium resolution mode (Δλ/λ = 4.3%). Polarised neutrons are generated by a Fe/Si supermirror (m = 3.8) that transmits neutrons in the spin down polarisation resulting in a cold neutron spectrum in the range of 2.5 Å < λ < 13.0 Å. Neutron RF spin-flippers operating at 236 kHz flip the polarisation of neutrons to the up spin state with 99.7 % efficiency. Pre- and post-sample guide fields maintain the polarisation of the neutrons during their passage through the instrument. Neutron counts were collected on 2-dimenstional ^3^He detector (Denex GmbH, Germany). Reflected beam spectra were collected at incident angles of 0.45˚ (0.72 mm slits) for 15 minutes, 1.40˚ (2.24 mm slits) for 15 minutes and 4.20˚ (6.72 mm slits) for 1 hour at each polarisation state. Direct beam measurements through silicon were collected under the same collimation conditions for each spin state. Data was reduced using the SLIM reduction package ([Nelson 2010](#_ENREF_8)) which stiches the three reflectivity profiles together at the appropriate overlap regions, re-bins the data to instrument resolution, subtracts a measured background and corrects for detector efficiency. A correction for the polarisation efficiency was not necessary, as the spin state of the reflected beam was not analysed. The final reflectivity profile is presented as a function of momentum transfer, Q, defined by:

$$Q=\frac{4\pi\sin\theta}{\lambda}$$

where θ is the angle of incidence and λ is the neutron wavelength.

Data was analysed using the MOTOFIT reflectivity analysis software ([Nelson 2006](#_ENREF_7)). A least squares fitting routine, which selects the best fit by minimising χ^2^ values between model and experimental data by varying the thickness, interfacial roughness and neutron scattering length density of each layer, was utilised. A genetic algorithm was used so that fits avoid falling into local χ^2^ minima and also so that constraints could be put on each parameter to be fitted. The model fitting of the reflectivity profiles yields information on the neutron scattering length density (nSLD) profile normal to the surface. The nSLD can be considered as the neutron reflective index and is a function of the chemical composition of each material according to

$$nSLD=N_{A}\sum_{i} \frac{p_{i}}{A_{i}}b_{i}$$

where N_A_ is Avogadro’s number, p_i_ the mass density, A_i_ the atomic mass and b_i_ the nuclear scattering length of component i. In neutron scattering the nuclear scattering length varies from element to element *and* from isotope to isotope. In this case the difference in scattering length between hydrogen (b_H_ = -3.74 🞨10^-5^ Å) and its isotope deuterium (b_D_ = +6.67 🞨10^-5^ Å) is particularly useful. By selective deuteration of components and choosing the correct isotopic contrasts different components of the biological layers can be highlighted or made ‘invisible’ to the neutrons. Each contrast is fitted simultaneously and, when changing between isotopic solvent contrasts, the physical structure of the biological layers is assumed to be stable. Therefore, when changing between contrasts, the thickness and roughness is kept constant and only the nSLD is allowed to vary. By using polarised neutrons of defined spin state an additional, magnetic, contrast is possible. Buried between the silicon wafer and the gold coating is a layer of Permalloy (80 % nickel / 20 % iron). The Permalloy not only acts to bind the gold to the silicon substrate but when magnetised is an additional source of scattering length density contrast. The sample is magnetised in a permanent magnetic field of ~10 mT and when a neutron reflects from this layer the reflectivity will depend on the neutron spin state. This gives two reflectivity profiles of differing contrast without affecting the biological layer. The two reflectometry profiles that arise from the spin-dependent reflection of the Permalloy provide an external reference from the isotopic substitutions in the biological layer to which the models can be constrained ([Holt et al. 2009](#_ENREF_3); [Kirby et al. 2012](#_ENREF_6)).

Error analysis was carried out by using a Monte Carlo resampling procedure on the best fit to the data sets as described previously ([Heinrich et al. 2009](#_ENREF_2); [Holt et al. 2009](#_ENREF_3)). At least N = 1008 synthetic data sets were produced by applying random Gaussian weighted deviations from the data based on the counting statistics of the real data. The synthetic data sets were analysed in the same manner as real data, outputting N variations for each parameter. The fits to the synthetic data were analysed by producing a frequency plot of the fitted values. The distribution of each parameter was statistically analysed with the parameter value being the midpoint of the 95 % confidence interval and the error defined as twice the standard deviation of the distribution.

**Supplementary Tables and Figures**

**Supplementary Table S1**: Analysis of the MALDI-TOF mass spectra to determine deuteration level of deuterated GGZctOmpA.

| **Mass of Hydrogenous fragment (m/z)** | **Amino acid sequence of fragment** | **Mass of corresponding deuterated fragment (m/z)** | **Percentage deuteration** |
| --- | --- | --- | --- |
| 1654.7303 | LGYPITDDLDIYTR | 1711.9594 | 63.93 % |
| 2601.0339 | NHDTGVSPVFAGGVEYAITPEIATR | 2688.3999 | 63.41 % |
| 2822.8918 | LGWSQYHDTGFINNNGPTHTREEF | 2902.2710 | 62.64 % |

**Supplementary Table S2:** The assembly of GGZctOmpA and thioPEG on gold for SPR experiments.

| **Sample** | **Contact time (sec)** | **Flow rate (μl/min)** |
| --- | --- | --- |
| SDS 1% v/v | 100 | 5 |
| Pause (PBS buffer) | 50 | 5 |
| GGZctOmpA | 300 | 5 |
| SDS 1% v/v | 180 | 5 |
| GGZctOmpA | 300 | 5 |
| SDS 1% v/v | 180 | 5 |
| GGZctOmpA | 300 | 5 |
| SDS 1% v/v | 180 | 5 |
| ThioPEG | 600 | 5 |
| SDS 1% v/v | 180 | 5 |
| ThioPEG | 600 | 5 |
| SDS 1% v/v | 180 | 5 |
| Pause (PBS buffer) | 200 | 5 |

**Supplementary Table S3:** The procedure for binding mouse monoclonal IgG to an array of GGZctOmpA and for cross-linking by SPR.

| **Sample** | **Contact time (sec)** | **Flow rate (μl/min)** |
| --- | --- | --- |
| mAb245 | 600 | 5 |
| Pause (PBS buffer) | 300 | 5 |
| Cross-linking reagent | 900 | 5 |
| 1 M Tris pH 6.8 | 900 | 5 |
| 100 mM HCl | 120 | 5 |
| Pause (PBS buffer) | 200 | 5 |

**Supplementary Table S4:** The procedure for binding of antigen (rNP) and secondary antibody (mAb108) by SPR.

| **Sample** | **Contact time (sec)** | **Flow rate (μl/min)** |
| --- | --- | --- |
| Pause (baseline) | 100 | 5 |
| rNP | 300 | 5 |
| Pause (dissociation) | 500 | 5 |
| mAb108 | 300 | 5 |
| Pause (dissociation) | 300 | 5 |
| 100 mM HCl | 120 | 5 |
| Pause (baseline) | 200 | 5 |


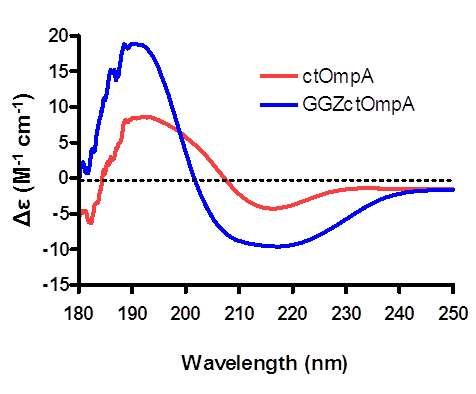


**Supplementary Figure S1:** CD spectra of ctOmpA (red) and GGZctOmpA (blue) which shows that GGZctOmpA successfully refolds from inclusion bodies. The CD signal has been normalised to take into account protein concentration and number of amino acids of each protein.


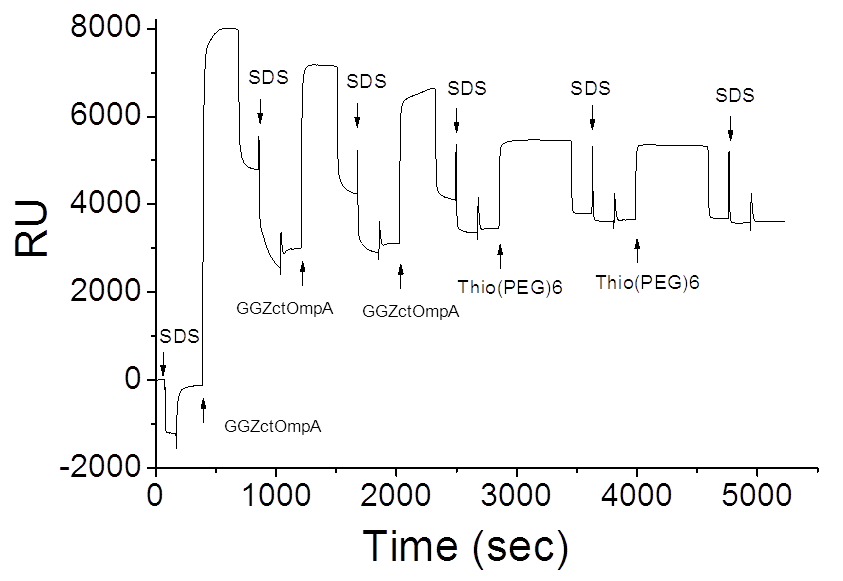


**Supplementary Figure S2:** An SPR sensogram of scaffold-protein surface assembly, three injections of GGZctOmpA and two injections of thioPEG, with 1 % SDS wash cycles between injections. Approximately 3550 RU of GGZctOmpA was deposited.


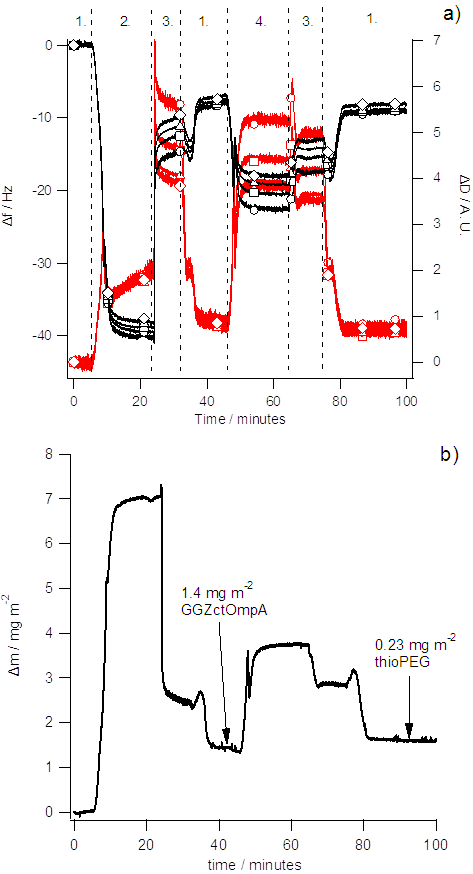


**Supplementary Figure S3:** a) QCM-D trace showing the change in frequency (black, left axis) and change in dissipation (red, right axis) for the fifth (○), seventh (□), ninth (△) and eleventh (◇) overtones for the creation of a GGZctOmpA array. The vertical dashed lines delineate between each stage of building the array with 1 = PBS buffer wash, 2 = 0.25 mg mL^-1^ GGZctOmpA incubation, 3 = 1% SDS wash to remove non-specifically bound material and 4 = 0.25 mg mL^-1^ thioPEG incubation. b) The fitted mass of the corresponding QCM trace.


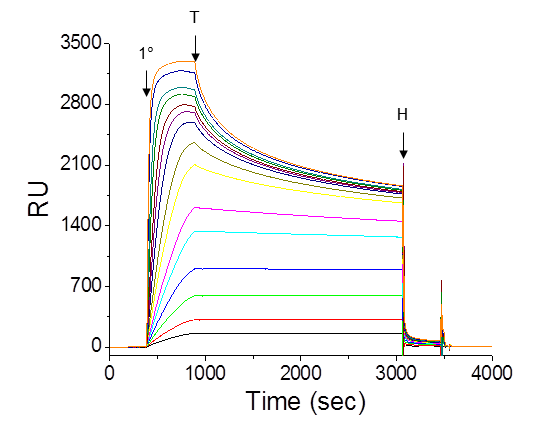


**Supplementary Figure S4:** The binding of mAb245 (concentration range 8 nM to 1 µM) to an array of GGZctOmpA with an association time of 600 seconds and a dissociation time of 2000 seconds at a flow rate of 5 µL min^-1^ with regeneration of the array between each concentration with 100 mM HCl. 1° - mAb injection, T – PBS buffer wash, H – regeneration with 100 mM HCl.


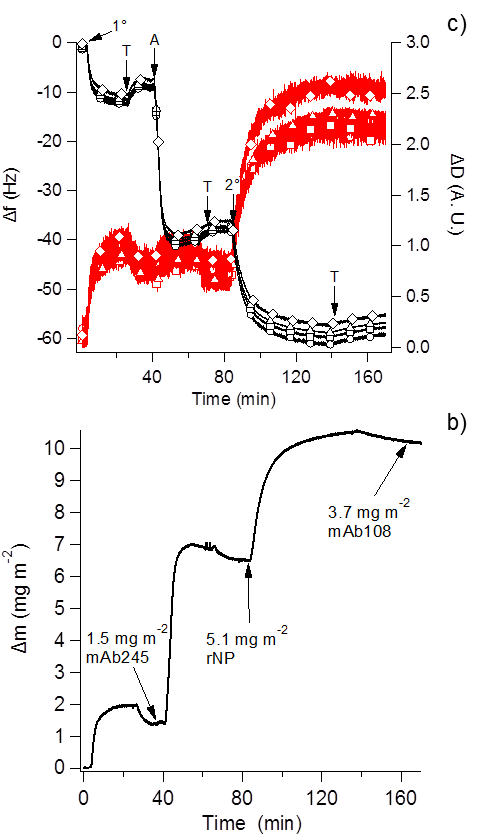


**Supplementary Figure S5:** c) QCM-D trace showing the change in frequency (black, left axis) and change in dissipation (red, right axis) for the fifth (○), seventh (□), ninth (△) and eleventh (◇) overtones for the binding of antibodies and rNP to an array of GGZctOmpA. d) The fitted mass of the corresponding QCM-D trace with the changes in mass at each step noted. 1° - 30 µg mL^-1^ mAb245 injection; T – PBS buffer wash; A – 10 µg mL^-1^ rNP injection, 2° - 30 µg mL^-1^ mAb108 injection.


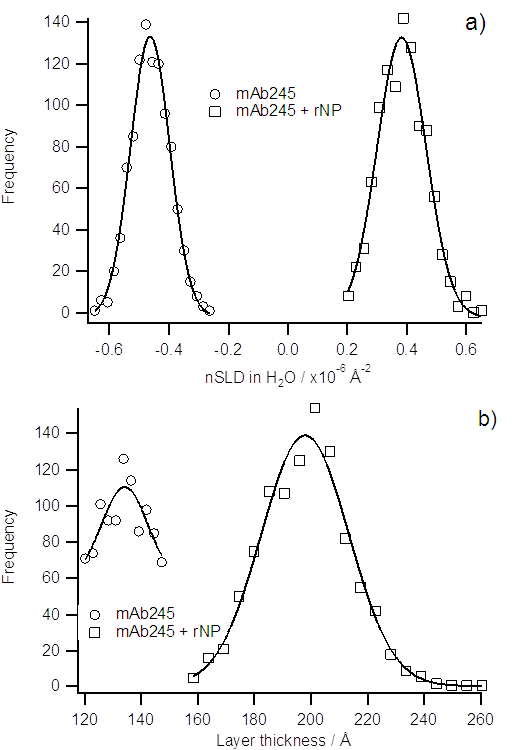


**Supplementary Figure S6:** The Monte-Carlo resampling results from the fits of the antibody/antigen layer. The frequency axis represents the number of times a particular result was obtained from 1008 fits. The results of the layer are for antibody only (○) and antibody plus antigen (□). The Gaussian fit is a guide to the eye only. a) Results from the nSLD in the H_2_O contrast and b) from the layer thickness.

**Supplemental Figure S7:** SPR sensogram of antibody and antigen binding to an array of ctOmpA (solid lines) and GGZctOmpA (dashed lines) at a flow rate 5 µL min^-1^. 1° - 30 µg mL^-1^ of mAb245, T- PBS buffer wash, A – rNP at 0 ng ml^-1^ (black), 100 ng mL^-1^ (red) and 1000 ng mL^-1^ (green), 2° - 30 µg mL^-1^ mAb108 injection, and H – regeneration with 100 mM HCl.


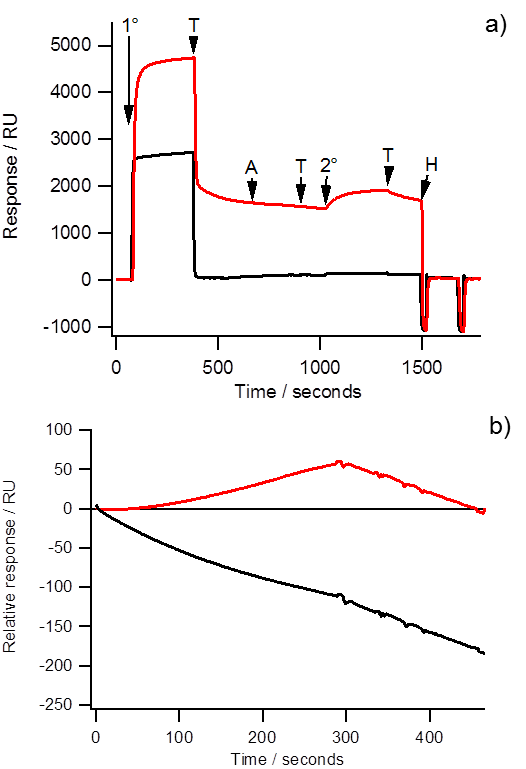


**Supplemental Figure S8:** a) SPR sensogram of mouse monoclonal anti-FLAG M2 IgG1 binding to an array of GGZctOmpA (red) and ctOmpA (black). A flow rate of 5 µL min^-1^ was used. 1° - 30 µg mL^-1^ mouse monoclonal anti-FLAG M2 IgG1, T – PBS buffer wash, A – 1 µg mL^-1^ rNP, 2° - 30 µg mL^-1^ mAb108, and H – regeneration with 100 mM HCl. b) A plot comparing the association and dissociation of 1 µg mL^-1^ rNP binding to mAb245 (red) and anti-FLAG M2 (black) both immobilised to an array of GGZctOmpA. The point of injection has been normalised to be time zero and a relative response of zero. The injection ends at 300 seconds after which a purely dissociation event is observed.


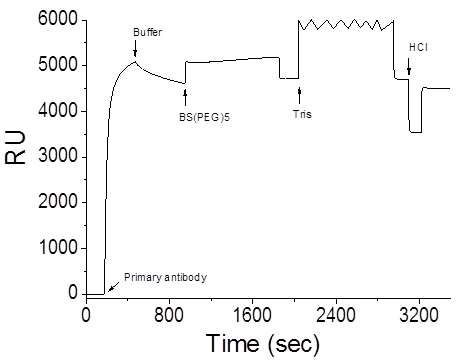


**Supplementary Figure S9:** SPR sensograms of cross-linking mouse monoclonal IgG to an array of GGZctOmpA with Bis(succinimidyl) penta(ethylene glycol) (BS(PEG)_5_). 30 μg mL^-1^ of antibody (mAb245) was incubated on the surface for 300 s at a flow rate of 5 μL min^-1^ this was followed by wash with PBS buffer. An incubation of 0.5 mM cross-linking reagent for 900 s was carried out to initialise cross-linking with the reaction terminated with 1 M Tris-HCl pH 6.8 followed by a wash of 100 mM HCl.

**Supplementary References**

Cooper MA, Singleton VT (2007) J Mol Rec 20:154-184

Heinrich F, Ng T, Vanderah DJ, Shekhar P, Mihailescu M, Nanda H, Losche M (2009) Langmuir 25:4219-4229

Holt SA, Le Brun AP, Majkrzak CF, McGillivray DJ, Heinrich F, Loesche M, Lakey JH (2009) Soft Matter 5:2576-2586

James M, Nelson A, Brule A, Schulz JC (2006) J Neut Res 14:91-108

James M, Nelson A, Holt SA, Saerbeck T, Hamilton WA, Klose F (2011) Nucl Instrum Meth A 632:112-123

Kirby BJ, Kienzle PA, Maranville BB, Berk NF, Krycka J, Heinrich F, Majkrzak CF (2012) Curr Opin Coll Interf Sci 17:44-53

Nelson A (2006) J Appl Crystallogr 39:273-276

Nelson A (2010) J Phys: Conf Ser 251:012094

Rodahl M, Hook F, Fredriksson C, Keller CA, Krozer A, Brzezinski P, Voinova M, Kasemo B (1997) Faraday Discuss 107:229-246

Saerbeck T, Klose F, Le Brun AP, Fuzi J, Brule A, Nelson A, Holt SA, James M (2012) Rev Sci Instrum 83:081301-081312
